# Filter feeding in Late Jurassic pterosaurs supported by coprolite contents

**DOI:** 10.7717/peerj.7375

**Published:** 2019-08-26

**Authors:** Martin Qvarnström, Erik Elgh, Krzysztof Owocki, Per E. Ahlberg, Grzegorz Niedźwiedzki

**Affiliations:** 1 Department of Organismal Biology, Evolutionary Biology Centre, Uppsala University, Uppsala, Sweden; 2 Institute of Paleobiology, Polish Academy of Sciences, Warsaw, Poland

**Keywords:** Filter feeding, Coprolites, Pterosaur, Palaeoecology, Ctenochasmatidae, Late Jurassic

## Abstract

Diets of pterosaurs have mainly been inferred from indirect evidence such as comparative anatomy, associations of co-occurring fossils, and functional morphology. Gut contents are rare, and until now there is only a single coprolite (fossil dropping), with unidentified inclusions, known. Here we describe three coprolites collected from a palaeosurface with numerous pterosaur tracks found in early Kimmeridgian (Hypselocyclum Zone) intertidal deposits of the Wierzbica Quarry, Poland. The specimens’ morphology and association to the tracks suggest a pterosaur producer. Synchrotron scans reveal numerous small inclusions, with foraminifera making up the majority of the identifiable ones. Other small remains include shells/carapaces (of bivalves, ostracods, and other crustaceans/arthropods) and bristles (some possibly of polychaete worms). The high density of the small shelly inclusions suggest that they were not accidently ingested, but constituted an important food source for the pterosaur(s), perhaps together with unpreserved soft-bodied animals. The combined evidence from the tracks and coprolites suggest a filter-feeding ctenochasmatid as the most likely tracemaker. If true, this significantly expands the bromalite record for this pterosaur group, which was previously only known from gastroliths. Moreover, this study also provides the first direct evidence of filter feeding in Jurassic pterosaurs and shows that they had a similar diet to the recent Chilean flamingo (*Phoenicopterus chilensis*).

## Introduction

Pterosaurs were a group of archosaurs that were the first among tetrapods to evolve powered flight (Unwin, 2003; Witton, 2013). They originated in the Late Triassic and constituted an important part of Mesozoic ecosystems until their extinction at the end of the Cretaceous (Unwin, 2003). During this period of time, pterosaurs adapted to diverse lifestyles, which is underlined by their large disparity, size variation, global distribution, and association to various depositional environments (Barrett et al., 2008; Henderson, 2010; Prentice, Ruta & Benton, 2011; Navarro, Martin-Silverstone & Stubbs, 2018).

Pterosaurs have been linked to piscivorous, carnivorous, insectivorous, herbivorous, durophagous, omnivorous, scavenging, and filter-feeding habits (Unwin, 2005; Osi, 2011; Martill, 2014; Witton, 2017; Zhou et al., 2017; Bestwick et al., 2018). Most of these dietary inferences are based on comparative anatomy, fossil associations, and functional morphology (Bestwick et al., 2018), but there are rare instances of direct evidence as well. Dietary gut and stomach contents from pterosaurs are dominantly represented by fish or unidentified vertebrate remains (Witton, 2017; and references therein). These are described from only a few localities (e.g., Solnhofen) and the genus *Rhamphorhynchus* has, by far, the most extensive record (Wellnhofer, 1975; Frey & Tischlinger, 2012; Witton, 2017).

Coprolites are relatively common fossils and they have the potential to yield a great deal of information about the palaeoecology of the producers (Hunt, Chin & Lockley, 1994; Chin, 2002; Qvarnström, Niedźwiedzki & Žigaite, 2016). Nevertheless, there is so far only a single specimen linked to a pterosaur producer (Hone et al., 2015). This fossil was found in conjunction with an articulated specimen of *Rhamphorhynchus* (with putative vertebrate remains in its gut) and contains hundreds of small spikes of unknown origin (Hone et al., 2015). Due to this relatively poor and selective record of direct dietary evidence, new discoveries have great potential to further increase our understanding of pterosaur ecology (Witton, 2017).

**Figure 1:**
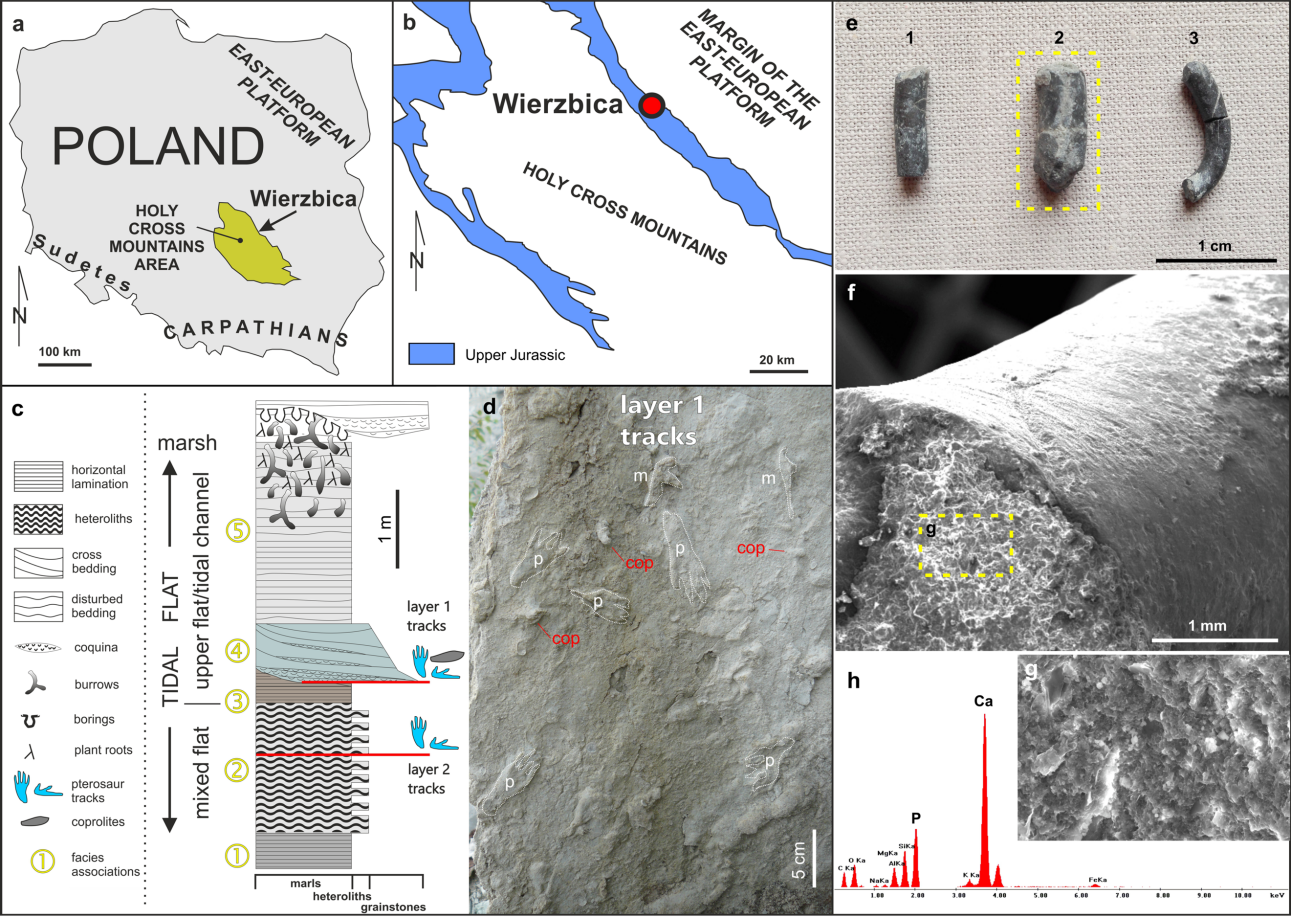
Details of the locality and coprolite specimens. (A) Map showing the location of the Wierzbica Quarry in Poland. (B) The location of the Wierzbica Quarry in a simplified geological map of the northern margin of the Holy Cross Mountains (based on Czarnocki, 1938; Urban & Gagol, 2008). (C) Detailed geological section of the tidal flat record with a consecutive succession of facies associations (1–5). The horizons with pterosaur tracks and coprolites are located at the bottom of facies association 4 (upper horizon with tracks) and within facies association 2 (lower horizon with tracks), (modified from Pieńkowski & Niedźwiedzki, 2005). (D) Details of the bottom of track layer 1 with record of pterosaur tracks (*m*–manus; *p*–pes) and coprolites (cop). (E) The studied coprolite specimens (MUZ PGI 1663.II.15a-c). (F, G) SEM images of coprolite matrix showing more or less irregular spheres and voids after bacterial activity. (H) EDS spectrum showing the geochemical composition of the coprolite matrix.

In this study, we analyze three coprolites (MUZ PGI 1663.II.15a-c) found in association with pterosaur tracks (Fig. 1) in a marginal marine setting, in order to identify the tracemaker and infer its diet. The specimens were analyzed using scanning electron microscopy (SEM) coupled with energy-dispersive X-ray spectroscopy (EDS) and propagation phase contrast synchrotron microtomography (PPC-SRµCT), a method which has been recently shown to be capable of non-destructively imaging entire coprolite contents of various sorts in three dimensions (Zatoń et al., 2017; Qvarnström et al., 2017; Qvarnström, Ahlberg & Niedźwiedzki, 2019a; Qvarnström et al., 2019b).

## Material and Methods

### The locality, palaeoenvironment and pterosaur tracks

The abandoned Wierzbica Quarry is located some 20 km south of the town Radom on the Mesozoic margin of the Holy Cross Mountains area of southern Poland (Fig. 1). Roughly 60 m of early Kimmeridgian carbonates outcrop in the quarry and these have been assigned to the Wierzbica Oolite and the Platy Limestone (Gutowski, 2004). The succession displays a shallowing-upward cycle, with open shelf facies in the bottom that pass into intertidal facies of the topmost part (Gutowski, 2004). This interpretation was confirmed and further elaborated by Pieńkowski & Niedźwiedzki (2005), who distinguished five facies/environmental units: (1) grey laminated marls—attributed to lagoonal/lower tidal flat facies; (2) white/grey heteroliths (flaser, wavy and lenticular bedding) composed of micritic limestones/marls indicating slack water conditions, and predominantly oolitic-organodetrital grainstones—attributed to a mixed tidal flat/intertidal environment; (3) laminated grey-brownish clayey marls with a strongly impoverished microfauna—attributed to an upper tidal flat, interfingering with; (4) cross-bedded, greenish/grey grainstones with redeposited shells, glauconite—attributed to a tidal channel; and (5) pelitic limestones and marls with disturbed bedding and numerous trace fossils—representing an upper tidal flat deposit. Pterosaur footprints occur in two layers; within facies unit (2) and in the interface of facies (3) and (4) (Fig. 1). The coprolites were found and collected from the latter.

The first report of pterosaur tracks in the locality was given by Pieńkowski & Niedźwiedzki (2005), who reported the occurrence of several isolated ichnites (which do not form trackways) on an intertidal palaeosurface in facies association 4 (Fig. 1). More recently, Elgh, Pieńkowski & Niedźwiedzki (2019) revisited the track record in Wierzbica, attributed the tracks to the ichnogenus *Pteraichnus*, and performed morphometric analyzes to narrow down the most likely producer(s) by linking the tracks to the pterosaur body fossil record. The results from the morphometric analyses taken together with occurrence data suggested that ctenochasmatoids, or possibly non-pterydactyloid monofenestratans or rhamphorhynchids, were the most likely trackmakers (Elgh, Pieńkowski & Niedźwiedzki, 2019).

### Phase-contrast synchrotron microtomography

The three coprolites (housed at MUZ PGI -Geological Museum, Polish Geological Institute-National Research Institute, Poland) were scanned using propagation phase-contrast synchrotron microtomography (PPC-SRµCT) at beamline ID19 of the European Synchrotron Radiation Facility (ESRF) in Grenoble, France. The fossils were scanned in vertical series of four mm, in half acquisition mode (the center of rotation was set at the side of the camera field of view, resulting in a doubling of the reconstructed field of view). The propagation distance (the distance between the sample on the rotation stage and the camera) was 2,800 mm. The camera was a sCMOS PCO edge 5.5 detector, mounted on an optical device bringing an isotropic voxel size of 6.36 µm. The camera was coupled to a 500-µm thick LuAG:Ce (Lutetium Aluminum Garnet activated by cerium) scintillator. The beam was produced by a W150 wiggler (11 dipoles, 150 mm period) with a gap of 51 mm and was filtered with 2.8 mm aluminum and 6 mm copper. The resulting detected spectrum had an average energy of 112 keV. Each sub scan was performed using 6000 projections of 0.05s each over 360 degrees.

The reconstructions of the scanned data were based on a phase retrieval approach (Paganin et al., 2002; Sanchez et al., 2012). Ring artefacts were corrected using an in-house correction tool (Lyckegaard, Johnson & Tafforeau, 2011). Binned versions (bin2) were calculated for fast processing and screening of the samples. The final volumes consist in stacks JPEG2000 images that were imported and segmented in the software VGStudio MAX version 3.1 (Volume Graphics Inc., Heidelberg, Germany).

### Scanning electron microscopy

One of the specimens was studied for microstructure and geochemistry using scanning electron microscopy (SEM) coupled with energy-dispersive X-ray spectroscopy (EDS). The specimen was attached to a stub and coated with platinum.

## Results

### Coprolite characteristics

**Figure 2:**
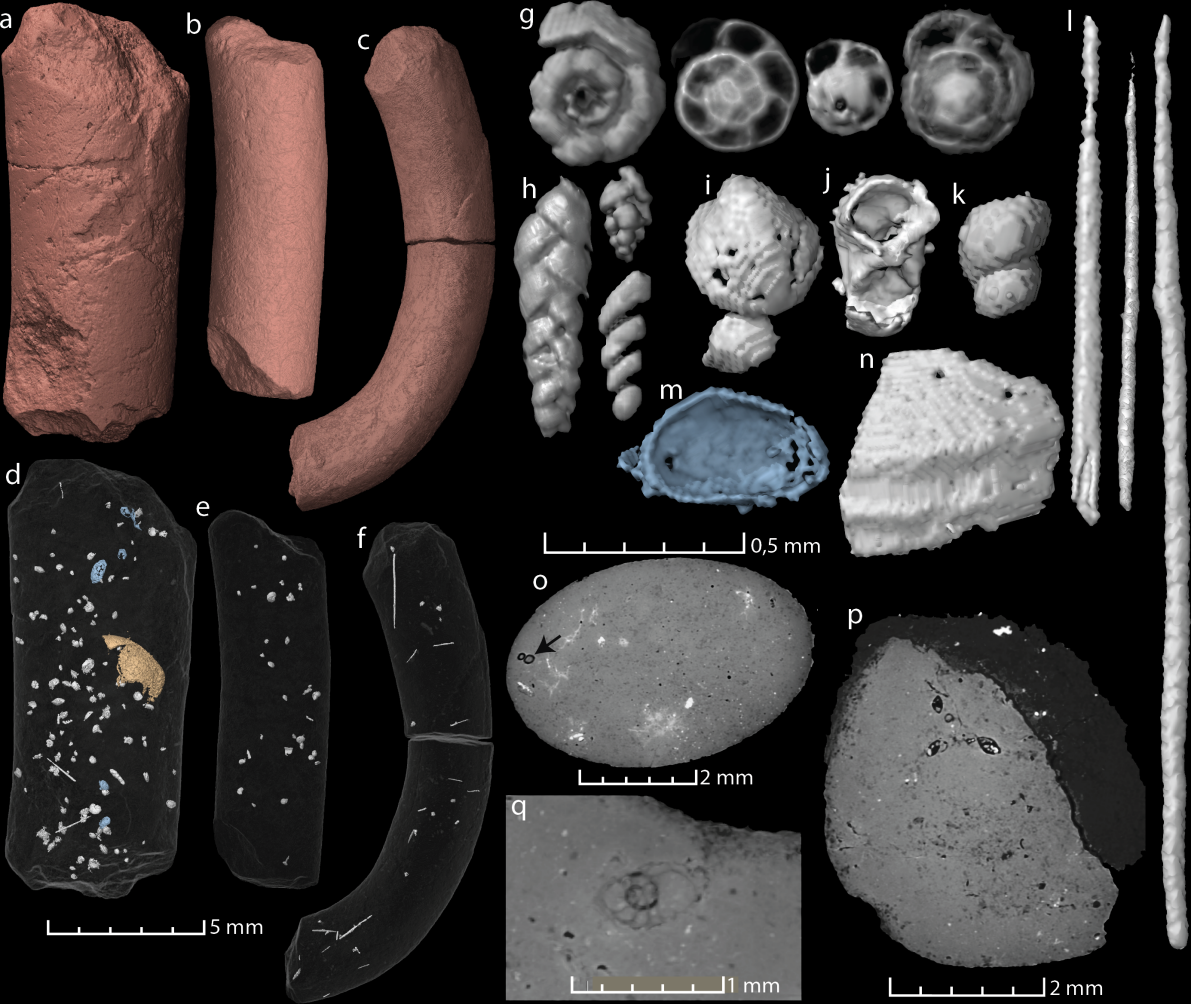
Virtual reconstructions and virtual thin sections of coprolites and inclusions. (A–C) 3D-models and gross morphology of the coprolites specimens. (D–F) Semi-transparent coprolites with foraminifera, bristles, gastropods, ostracods (blue), and one large enigmatic structure (yellow/orange) highlighted. Note that each coprolite contains many small unidentified inclusions and cracks, which are not included in these models. (G) Examples of spiral foraminifera. (H) Uni-, bi- and triserial foraminifera. (I) Bivalve. (J) Crustacean body (amphipod?). (K) Part of a gastropod, same as seen in (O). (L) Examples of bristles. (M) Ostracod carapace. (N) A relative large shell fragment. (O) Gastropod fragment in a virtual thin section. (P) A virtual thin section of coprolite matrix with several foraminifera in the topmost part. (Q) Virtual thin section of coprolite matrix and a relatively large spiral foraminifera. Images (A, D, G–J, P) and the two bristles to the left in (L) derive from specimen MUZ PGI 1663.II.15a; (C, F, N) and the rightmost bristle in (L) from specimen MUZ PGI 1663.II.15b; and (B, E, K) from specimen MUZ PGI 1663.II.15c.

All three studied coprolites are elongated, cylindrical, non-spiral, and smooth (Figs. 1 and 2, Table 1). They are of somewhat comparable sizes, although specimen MUZ PGI 1663.II.15a has a larger diameter than the other two. The SEM study revealed a matrix structure characteristic for phosphatic coprolites, with more or less irregular spheres and voids after bacterial activity. The coprolite composition was characterized using EDS, which showed calcium and phosphate as the dominant components. The scans revealed some internal differences; specimen MUZ PGI 1663.II.15a has a homogenous matrix with abundant inclusions, whereas the other two specimens have more heterogeneous matrices with many unidentifiable remains and voids. Foraminifera are present in all coprolites but are most abundant in MUZ PGI 1663.II.15a, which contains over 100 specimens (Fig. 2; Table 1). These are of several morphologies including spiral (trocho- and planispiral; Figs. 2G, 2P, 2Q), serial (uni-, bi-, and triserial; Fig. 2H) and possibly single-chambered forms. Rarer inclusions comprise remains of ostracods, bivalves, gastropods, and crustaceans as well as possible bristles from polychate worms that are especially abundant in specimen MUZ PGI 1663.II.15b (e.g., Figs. 2F, 2I–2O). A major part of the extremely abundant inclusions, however, are near impossible to identify due to their fragmented and/or poorly preserved state.

**Table 1:** List of the analyzed coprolite specimens.

**Specimen**	**Length** × max. width	**Inclusions**	**Comments**
MUZ PGI 1663.II.15a	14,3 × 6 mm	>100 foraminifera, ostracods, bristles (possibly from polychaete worms), carapaces of small crustaceans, a gastropod, a bivalve, one large vaulted structure (possibly a poorly preserved shell), and unidentified shell fragments.	
MUZ PGI 1663.II.15b	18 × 3,2 mm	>10 foraminifera, abundant bristles, fragmented small shells.	Coprolite curved.
MUZ PGI 1663.II.15c	12,2 × 3,7 mm	>20 foraminifera, a gastropod, many small voids and silt-sized grains. Some internal cracks.	

## Discussion

### The producer and ecological inferences

The overall shape, internal structure, and phosphatic composition of the specimens (Figs. 1 and 2) imply that they are coprolites. Cylindrical and elongated coprolites are known from various vertebrate and invertebrate producers (e.g., Häntzschel, El-Baz & Amstutz, 1968; Hunt & Lucas, 2012). The faecal pellets of the latter, however, are commonly smaller than the specimens from Wierzbica (Häntzschel, El-Baz & Amstutz, 1968), rendering a vertebrate producer probable. The preservation of the pterosaur tracks in the intertidal environment indicates a fast burial, implying that the droppings preserved on the same surface were deposited at a similar time to the track formation. Furthermore, both the size and shape of the coprolites are very similar to the so far only described pterosaur coprolite (Hone et al., 2015) and the coprolite sizes match those of the footprint maker(s) at Wierzbica (Elgh, Pieńkowski & Niedźwiedzki, 2019). Taken together, these facts suggest that pterosaurs were most likely scat producers.

Given the concentrations of foraminifera, minute shelly animals and probable polychaete bristles in the coprolites, it seems probable that these organisms were deliberately targeted rather than accidentally ingested. A reasonable explanation for how a pterosaur big enough to have produced the droppings effectively could have captured such small prey is through filter feeding. The modern filter-feeding Chilean flamingo (*Phoenicopterus chilensis*) has been shown to produce droppings rich in foraminifera (and also copepods and polychaetes), in similar to the coprolites described here (Tobar et al., 2014).

A glance at the pterosaur body fossil record reveals that several potential filter feeders belonging to the group Ctenochasmatidae were around at this time, both regionally (e.g., *Ctenochasma* and *Gnathosaurus*) and elsewhere (*Liaodactylus*); see Zhou et al. (2017) for phylogenetic interrelationships and inferred ghost lineages of the taxa. Filter feeding in these Jurassic ctenochasmatids is supported by their long rostra and many slender, closely-spaced teeth (Wellnhofer, 1970; Bennett, 2007; Zhou et al., 2017). It has been argued that they were not filter feeders, or at least not exclusively so, with reference to the more specialized jaw apparatus (a sieving basket consisting of many long, slender teeth sitting in upcurved dentaries) found in the Cretaceous taxon *Pterodaustro* (Sanderson & Wassersug, 1993; Witton, 2013). However, evidence from computed models of digitized pterosaur skulls support that *Ctenochasma* and *Gnathosaurus,* in similar to *Pterodaustro*, had very low bite forces, but fast closing jaws, which would facilitate feeding on small evasive prey/filter feeding (Henderson, 2018).

The combined evidence of the pterosaur tracks, which are possibly ctenochasmatid (Elgh, Pieńkowski & Niedźwiedzki, 2019), and the coprolites, which were most likely made by a filter feeder, leads us to conclude that filter-feeding ctenochasmatids were probable producers of both tracks and droppings. Our findings significantly expands the bromalite record for this pterosaur group, which was previously only known from gastroliths found in *Pterodaustro* (Codorniú, Chiappe & Cid, 2013), and lends further support to filter-feeding among these Jurassic taxa.

**Figure 3:**
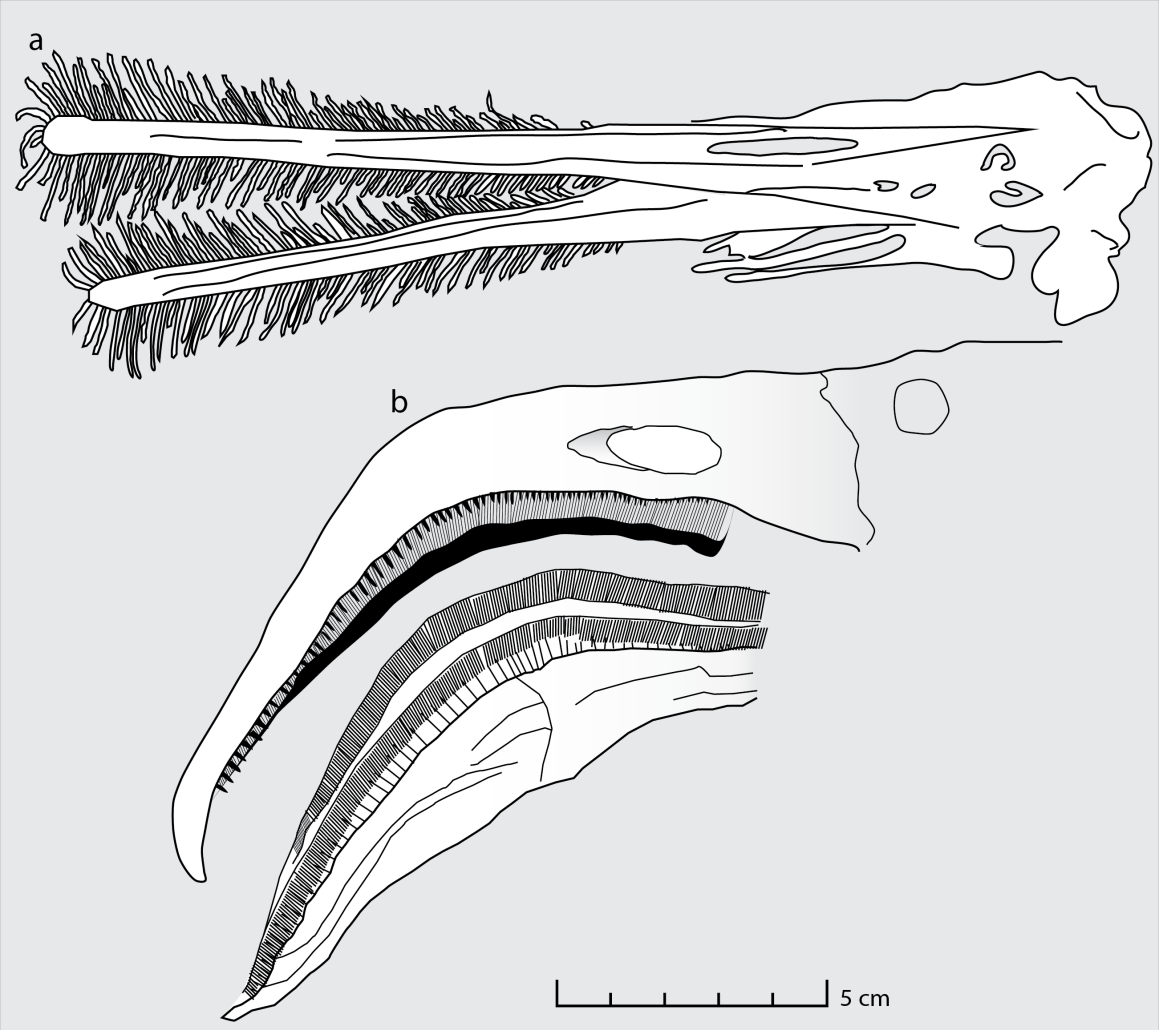
Feeding apparatus of *Ctenochasma elegans* and *Phoenicopterus chilensis*. Schematic drawing of (A) the jaws and teeth of *Ctenochasma elegans* (redrawn from Bennett, 2007) and (B) the beak of the recent Chilean Flamingo (redrawn from Mascitti & Kravetz, 2002).

Moreover, the mesh size implied by the size of the coprolite inclusions can be matched not only in flamingos but also in *Ctenochasma*. The functional sieve in the Chilean flamingo has been shown to range from 80 to 959 µm across the proximal-distal axis of the beak (Mascitti & Kravetz, 2002). Most of the Wierzbica coprolite inclusions fall within this size range (most specimens being around 300 µm in size) indicating a similar mesh size. Such a small mesh-size was probably present in adult specimens of *Ctenochasma elegans*, evidenced by a tooth-spacing of 7.36 teeth/cm in one specimen (Bennett, 2007; Fig. 3). Comparable mesh sizes and feeding environments might explain the similar dietary contents of the Chilean flamingo and these ctenochasmatid pterosaurs. It should be noted that the Jurassic ctenochasmatids were perhaps not capable of active pumping, in contrast to recent flamingos, and possibly *Pterodaustro* (e.g., Sanderson & Wassersug, 1993).

The laminated grey/brownish clayey marls from facies association 3, where the traces occur (track layer 1), revealed strongly impoverished benthic microorganisms, composed of scarce foraminifera tests (*Spirilina* sp. and *Lenticulina* sp.), rare ostracod carapaces and broken echinoid spines. Collectively, these finds point to relatively shallow water condition with marine benthic association (Pieńkowski & Niedźwiedzki, 2005) for the pterosaur track and coprolite horizon. The high abundance and disparity of the foraminifera in the coprolites further suggest that they were strongly targeted by the coprolite producers and/or that they derive from another feeding environment.

The size of the foraminifera indicates a high abundance of benthic forms in the coprolites, as pelagic ones are normally less than 100 µm in size (Armstrong & Brasier, 2005). However, there are possibly some small, but not very well-preserved specimens in the coprolites as well. The fact that the shells and tests at all are present in the coprolites also provides some clues about the digestive system of the producers. Calcitic, and likely aragonitic, material is typically completely dissolved in the digestive system of recent crocodiles, leaving keratinous structures but no bones or shells in their faeces (Fisher, 1981; Milàn, 2012). However, bones of various degree of etching are commonly found in coprolites of other archosaurs such as theropod dinosaurs (Thulborn, 1991; Chin et al., 1998; Qvarnström, Ahlberg & Niedźwiedzki, 2019a). It appears therefore that the pterosaurs at Wierzbica, in similar to some archosaurs, had shorter food retention time and/or weaker stomach acids than seen in recent crocodiles.

Specimen MUZ PGI 1663.II.15a was likely produced by a larger individual than that/those which produced the other two coprolites since animal size and diameter of faeces positively correlate (e.g., Milàn, 2012). Size variation is also seen in the footprint record of Wierzbica (Elgh, Pieńkowski & Niedźwiedzki, 2019), altogether suggesting the presence of a pterosaur flock with individuals of different ontogenetic stages and/or sympatric species. The higher relative abundance of foraminifera in the biggest coprolite might suggest an ontogenetic switch to a more specialized filter feeding in adults, whereas younger individuals relied more on eating soft-bodied organisms from the sediments. Such a dietary switch is consistent with the addition of more teeth across the ontogenetic series of *Ctenochasma elegans* (Bennett, 2007).

## Conclusions

The pterosaurs that roamed the intertidal environments in Wierzbica were most likely filter feeders with a functional sieve optimizing capture of prey with a diameter of around 300 µm. Their diet, including foraminifera and other small invertebrate prey, was much like that of the Chilean flamingo. This study presents the first direct evidence of filter-feeding in pterosaurs and we hope that future identifications of coprolites produced by pterosaurs may greatly improve our understanding of their palaeoecology.

## Additional Information and Declarations

### Data Availability

The following information was supplied regarding data availability: The reconstructed stacks of jpeg2000 images of all coprolites are publicly available in ESRF’s paleontological microtomographic database: http://paleo.esrf.eu/index.php?/category/2410. The specimens are stored at MUZ PGI -Geological Museum, Polish Geological Institute, National Research Institute, Poland. Specimen numbers: MUZ PGI 1663.II.15a, MUZ PGI 1663.II.15b, and MUZ PGI 1663.II.15c.
